# Visceral Therapy and Physical Activity for Selected Dysfunctions, with Particular Emphasis on Locomotive Organ Pain in Pregnant Women—Importance of Reducing Oxidative Stress

**DOI:** 10.3390/antiox11061118

**Published:** 2022-06-05

**Authors:** Małgorzata Wójcik, Grażyna Jarząbek-Bielecka, Piotr Merks, Dawid Luwański, Katarzyna Plagens-Rotman, Magdalena Pisarska-Krawczyk, Małgorzata Mizgier, Witold Kędzia

**Affiliations:** 1 Department of Physiotherapy, Faculty of Physical Culture in Gorzów Wielkopolski, Poznań University of Physical Education, 61-871 Poznan, Poland; 2 Division of Developmental Gynaecology and Sexology, Department of Perinatology and Gynaecology, Poznań University of Medical Sciences, 61-758 Poznan, Poland; grajarz@o2.pl (G.J.-B.); dawidluwan@gmail.com (D.L.); witold.kedzia@poczta.fm (W.K.); 3 Department of Pharmacology and Clinical Pharmacology, Faculty of Medicine, Collegium Medicum, Cardi-nal Stefan Wyszyński University, 01-938 Warszawa, Poland; p.merks@uksw.edu.pl; 4 Institute of Health Sciences, Hipolit Cegielski State University of Applied Sciences, 62-200 Gniezno, Poland; plagens.rotman@gmail.com; 5 Nursing Department, President Stanisław Wojciechowski State University of Kalisz, 62-800 Kalisz, Poland; magmp@op.pl; 6 Department of Dietetics, Faculty of Physical Culture in Gorzów Wielkopolski, Poznań University of Physical Education, 61-871 Poznan, Poland; m.mizgier@diaeteticus.pl

**Keywords:** visceral treatment, physical activity, pregnancy, back pain, oxidative stress

## Abstract

Movement is a physiological phenomenon and a fundamental aspect of the living human body in a global context (e.g., musculoskeletal system function) and local one (e.g., visceral system function). The local activity of the body is expressed in the rhythm of pulsations, peristalsis and vibrations. Visceral therapy supports movement, articulation and tissue rhythm. The use of visceral treatment for pain is complementary and is relevant for pregnant women. Maintaining the mobility and motility of internal organs by means of visceral techniques can regulate anatomical relations and physiological processes within the urogenital diaphragm. The role of physical activity is also important. A scoping review was conducted to analyze the relevant literature on pain in pregnant women, the role of visceral therapy in pregnant women and oxidative stress. Eligible articles presented aspects of the occurrence of pain in locomotive organs in pregnant women, the use of visceral therapy in pain management, and the reduction of oxidative stress. The use of visceral therapy and physical activity in the treatment of pain is complementary and also important for pregnant women, and so may have an effect on reducing oxidative stress in pregnant women.

## 1. Introduction

Pain in the locomotive organs is very common and also affects pregnant women. It turns out that the right course of action with postural and biomechanical factors prevents or reduces multiple complaints and pain, which means that pregnant women can avoid them. It is also possible to reduce the risk of serious issues such as pubic symphysis disorder, sacroiliac joint pain and sciatic neuralgia. The right mechanics of the body (particularly of the pelvis) has a positive influence on the course of the delivery and physical condition of a newborn baby [1]. 45–56% of pregnant women suffer from lower back and pelvic pain [2,3], of which about 20% concern the pelvic girdle [4,5]. They occur between weeks 12 and 18 of pregnancy (as observed in 62% of women), their prevalence increases between weeks 24 and 30 of pregnancy, and intensifies with each subsequent pregnancy [6]. Pregnancy is well-known to increase oxidative stress, a phenomenon generated by a normal systemic inflammatory response, resulting in high amounts of circulating reactive oxygen species (ROS). The major source of ROS during pregnancy is the central organ that regulates this condition, i.e., the placenta [7]. In this regard, increased oxidative stress seen in pregnancy could lead to potential tissue damage [8,9]. Increased oxidative stress is counterbalanced by an increase in the synthesis of antioxidants [10]. When oxidative stress exceeds the antioxidant defense in the placenta, oxidative damage could propagate to distal tissues. The occurrence of pain sensations in the musculoskeletal system has been shown to be related to the occurrence of oxidative stress [11].

Interdisciplinary collaboration in the area of practical treatments by gynaecologists, physiotherapists and osteopaths in pregnant women has indicated the effectiveness of those treatments which improve the quality of life, such as by eliminating pain in the muscular-skeletal structure through visceral therapy or physical activity aimed at reducing oxidative stress. However, the practice of interdisciplinary care of pregnant women is founded in scientific research that explains the links between the aspects raised by the authors. For this reason, the authors decided to conduct a review of the literature on the topic.

## 2. Methods

In the article, the authors consider the following aspects: pain in the locomotive system in pregnant women, the use of visceral therapy, the importance of physical activity and oxidative stress. The authors reviewed the literature databases in Pubmed, Medline and Embase in order to find link between these aspects in relation to pregnant women. The authors examine these aspects based on the available literature.

## 3. Oxidative Stress

Homeostasis of the body and its proper functioning are determined by an oxidative-antioxidative balance. Disorders in this balance are related most often to the overproduction of reactive forms of oxygen that cause oxidative stress. This stress underlies the development and course of many civilization diseases [12,13,14,15,16,17,18]. Environmental pollution, chronic stress, low levels of physical activity and improper nutrition are determinants for producing an increased amount of free radicals. The destructive activity of ROS consists in the oxidation of cell components, resulting in damage and disorders of physiological functions. Free radicals change the structure and functions of proteins. They also destroy the structure of DNA. Over the long process of evolution, the human body developed the ability to use oxygen as a source of energy [19,20,21,22,23].

Although life as such without oxygen is not possible, oxygen derivatives can also pose a threat. Reactive forms of oxygen are chemically active particles that can do a lot of harm in the human body, and their excess is called oxidative stress. Additionally, when the balance between the amount of free radicals and neutralizing antioxidants is upset in the body, this can lead to many diseases and faster aging. Free radicals are created during respiration—oxygen provides us with energy, but a portion of its particles are harmful. Each oxidant has a free unpaired electron and seeks a cell from which it can take an electron to pair up with. In this way, it damages healthy cells (proteins, fats, DNA). The protective shields for cells are antioxidants, whose task is “to give up” a free electron to the oxidant, neutralizing the free radicals and restoring proper functioning in the organism [24,25,26,27]. A disbalance between free radicals and antioxidants is called a state of oxidative stress [28,29] and can lead to atherosclerosis, stroke, cardiac infarction, visual disturbances and also coronary disease, among others. It also has a significant influence in the faster aging of the skin and even increases the risk of melanoma developing. Furthermore, oxidative stress also damages neurons in the brain, producing Parkinson’s and Alzheimer’s disease. An overly large amount of free radicals in the body also leads to damage to genetic material, and for this reason we are more exposed to cancers (not only of the skin).

Since the late 1980s, the term oxidative stress has appeared in the scientific literature, even though the processes related to this state had been a subject of interest for scientists much earlier [29]. Currently, over 490,000 links related to this issue can be found in internet searches. Oxidative stress occurs in the human body when the balance between pro-oxidative and antioxidative processes is disturbed. The activation of prooxidative processes can be caused by the excessive production of reactive forms of oxygen and nitrogen (RONS, *reactive oxygen and nitrogen species*) and also by an insufficient amount of antioxidants [30]. If such a state is short, the protective mechanisms of the body are stimulated and balance is restored. In the case of long-lasting oxidative stress, damage to cells occurs, primarily as a result of the oxidation of lipids, carbohydrates, proteins and nucleic acids [31]. Sensations of pain in the musculoskeletal system have also been shown to be related to the occurrence of oxidative stress [11].

## 4. Mechanisms and Molecular Factors Which Can Justify the Use of Exercise and Exercise-Based Rehabilitation Pre- and Post-Labor

Moderate exercise among reproductive-age women and during pregnancy affects the mother’s health and reduces the risk of complications related to fetal development and the occurrence of chronic diseases in children later in life [32]. Moreover, appropriate exercise is a key instrument in the management of chronic conditions associated with inflammation and oxidative stress (OS) [33,34]. OS and the excessive production of reactive oxygen species (ROS) in the body coexist in many conditions with low-grade chronic inflammation [35,36]. Many studies also indicate that physical activity may induce an inflammatory process and OS. However, an effect related to physical activity depends on the intensity and duration of exercise.

The anti-inflammatory effects of exercise are well described [33,34,37], but the mechanisms that trigger an inflammatory response and tissue damage are not fully explained in the literature. Exhaustive and long exercise may initiate the release of inflammatory cytokines and ROS, which cause muscle and tissue damage [38]. Intensive and prolonged physical activity can speed up the accumulation of ROS through the upregulation of energy production, mitochondrial electron transport chain leakage and the NADPH oxidase production of superoxide [39].

The mechanisms that trigger inflammatory response and tissue damage are not fully explained in the literature. The researchers suggest that during exhaustive exercise, stress and dehydration cause hypoperfusion of the internal organs (liver, kidney, and intestines) [40], consequently augmenting inflammatory responses and the production of proinflammatory cytokines such as Tumor Necrosis Factor-alfa (TNF-alfa) and interleukins: IL-1B, IL1ra, IL-1 alfa, IL-6, IL-8, IL-10 [38].

Taking into account the molecular mechanisms, we already know that OS induces the activation of transcription factors, for example, nuclear factor-kappa B (NF-κB). This factor regulates pro-inflammatory gene expression [38] and interacts in cells with the production of proinflammatory cytokines such as TNF-alfa and IL-6 [41]. The main role in developing OS and the inflammation process is played by mitochondria. However, on the other hand, an increased ROS load leads to a compensatory increase in the activity of non-enzymatic and enzymatic antioxidants [12,41,42].

Some molecular mediators can stimulate an antioxidant and anti-inflammatory response capable of assuring the attenuation of OS. There are studies describing exercise-related antioxidant effects and mediators such as nuclear factor (erythroid-derived 2)-like 2 (NrF2) [43] or deacetylase sirtuin 1 (SIRT1) [44]. NrF2 is a crucial factor in maintaining redox homeostasis. It is engaged in controlling antioxidant enzymes and pro-inflammatory gene expression [43]. Sirt1 mediates the response to OS by inducing the expression of antioxidant enzymes such as superoxide dismutase (SOD) and catalase (Cat). It has been proven that moderate exercise promoted Sirt1 activity in rats and induced an increased expression of antioxidant enzymes, such as SOD and Cat [44]. Further research should be directed toward looking for a non-harmful therapeutic method that suppresses the NF-κB signal pathway, promotes Sirt1 and activates the Nrf2/ARE (antioxidant response element) pathway [38].

The cardiovascular system affects the course of oxidative stress in the human body. In turn, physical activity has an impact on the cardiovascular function. Physical exercise in water has been shown to significantly reduce oxidative stress [45]. The application of photobiomodulation therapy prior to exercise plays an important antioxidant role by reducing exercise-induced oxidative stress and consequently increasing physical performance and improving post-exercise recovery [46]. High resistance inspiratory muscle strength training, a novel and effective training method, improves arterial tone and endothelial function in adults [47].

Oxidative stress is associated with hypertension, which may occur in menopausal women. Non-pharmacological treatment of grade 1 and 2 hypertension may include moderate aerobic exercise [48]. Resistance training can be another form of antioxidant system enhancement in women of all ages [49]. Another study showed that 12 weeks of simultaneous body modulation for women who performed resistance training and consumed whey protein positively affected antioxidant enzyme activity and oxidative stress markers [50]. In older women in particular, an imbalance between antioxidants and prooxidants is associated with hypertension. Low-intensity aerobic exercise would be a good option to eliminate oxidative stress [51]. Oxidative stress also affects muscle strength and physical activity. Moderate-intensity exercise in women has been shown to increase muscle strength and flexibility and to improve gait speed, in addition to its effect of reducing oxidative stress [52]. Physical activity during pregnancy is associated with improved fitness and a reduction in ailments during pregnancy, and it therefore influences the course of pregnancy and delivery in a positive way.

## 5. Locomotive Organs in Pregnant Women

The biomechanical balance changes during pregnancy as a result of multiple postural changes. Pregnancy changes the biomechanical balance of women. Although this should not cause pain in the locomotive organs, this is often the case in pregnant women. It is worth emphasizing that maintaining flexibility and elasticity of the spine, the pelvic girdle and the shoulder girdle allows the body to adjust to dynamic changes during pregnancy [1]. Inflexible structures surrounding and supporting the uterus can transmit forces onto the wall of the uterus and thus onto the growing baby. There is a bilateral relationship between the fetus position and uterine tone, sprains of uterine ligaments, and the biomechanics of the pelvis and the spine [1]. The thoracolumbar fascia largely impacts intra-abdominal pressure and thus contributes to the proper functioning of the lumbar and pelvic region [53]. The diaphragm, the iliopsoas muscle, the iliac fascia, the superficial fascia of the abdomen, the transversalis fascia, the fascia of the urogenital diaphragm and the presacral fascia are also essential elements of the fascial system [54].

A very important factor that affects tension in the fascial system is the curvatures of the spine (cervical and lumbar lordosis as well as thoracic kyphosis) that manifest an interdependent biomechanical relationship [55]. As pregnancy progresses, a change in the pattern of muscular coordination can occur, which may cause pain in the spine [56].

As mentioned above, pregnancy is accompanied by multiple postural changes. In the first trimester, the pelvis is slightly tilted backward by the pressure of moving viscera. Initially, the uterus presses on the bladder and the fundus of the uterus. As the uterus grows, the pressure moves to the small intestine and the sigmoid [1]. The posterior pelvic tilt increases the tone of the hip flexors, the lumbar erector spinae muscle and abdominal muscles (in the first trimester, the belly starts changing its shape). As the uterus expands, intraperitoneal organs (the intestinal column), i.e., the small intestine, the large intestine, the stomach and the liver, move up and place pressure on the diaphragm, while the thoracolumbar region is pulled into an upright position.

Increased kyphosis and the lower site of cervical lordosis result´ from breast growth in the first trimester of pregnancy, which can cause tension in the superior thoracic aperture. At the end of the second trimester, the pelvis begins to change its position from a posterior to an anterior tilt, increasing lumbar lordosis, causing tension in the linea alba and a feeling of tension in the lower part of the sternum and the epigastrium. The abdominal cavity changes in shape, expanding in the posterolateral region and causing tension in the arch ligaments of the diaphragm, rib joints, intercostal muscles, thoracolumbar fascia (rich in nociceptors), and the quadratus lumborum muscles. In the third trimester of pregnancy, women can exhibit a swayback posture, where the weight of the body lies on the pubic symphysis and abdominal muscles, often behind the pubic symphysis on the pelvic floor and ligaments [1].

The third trimester of pregnancy is a period of increased lumbar lordosis, and towards the end of the pregnancy or after delivery, the thoracolumbar region may flatten [57,58]. Changes in the volume of the enlarging uterus cause changes in pressure difference within the chest, which is divided into two parts by the diaphragm (above the diaphragm—the lungs, below—the viscera). The consequence of this pressure difference can be pain [1]. It is essential to perceive the body of a pregnant woman as a whole and to understand the relationships between the body cavities and the locomotor system.

The developing pregnancy also causes restrictions in spinal mobility in the sagittal and frontal planes. Another change is the increased support quadrilateral of the feet. These changes occur to reduce the impact of weight increase during pregnancy [58]. Movements in the shoulder and hip girdle as well as in the lower limbs adjust to changes in the spine, causing changes in the gait pattern, where changes in the rotation between the shoulder girdle and the hip girdle can be observed. While walking, the coordination between the rotation of the pelvis and the chest is very important. Pain in the pelvic region can result from an improperly coordinated rotation between the chest, shoulder girdle and hip girdle [59,60]. With a change in the gait cycle and the mechanics of the lower limbs, the tension in the soft tissues in the pelvis, the abdomen and the lower limbs will change.

Apart from the changes in the locomotive system during pregnancy, the mechanics of breathing also change as a consequence of changes in the shape of the chest. The upward shift of the diaphragm reduces the expiratory reserve volume and the residual volume of the lungs [61,62]. This is caused by changes in the dependencies between the chest and the pelvis during pregnancy. Pharmacology, kinesiotherapy, physiotherapy and exercise are used to treat pain in the locomotor organs in pregnant women.

Physiotherapy and exercises help with pain syndromes associated with the increased tension of muscle groups. It is recommended that one use safe and non-invasive manual techniques, transcutaneous nerve stimulation, acupuncture, autogenic breathing training, and aromatherapy [63,64].

Massage and techniques used to stretch muscles and other contracted and tense tissues have a relaxing effect and reduce pain by activating the transmission of nerve impulses through the A-fibers (blocking pain transmission) and/or stimulating endorphin secretion. The following muscle stretching techniques are used: post-isometric relaxation, reciprocal inhibition and myofascial release [65,66].

In kinesiotherapy, segmental stabilization exercises are used. The first stage involves segmental control exercises in the closed kinematic chain that integrates the functioning of the local muscles with the segmental transfer of gravitational forces. The best combination of activities in local (in pregnant women—paraspinal) and anti-gravity muscles is achieved through exercises with the body load and a gradually increasing force of gravity. Then, segmental control exercises in the open kinematic chain and functional exercises are implemented [67,68].

Physical activity as a preparation for delivery and rehabilitation in the postpartum period are recommended in order to improve quality of life and prevent pain [69,70,71]. A less common non-invasive and non-pharmacological method to reduce pain and tension in soft tissues is osteopathic visceral treatment. Santos et al. showed that visceral manipulations combined with a physiotherapeutic program improved mobility in the lumbar spine [71]. Visceral techniques can successfully be used for non-specific pain in the cervical spine. In the case of the liver and spleen, these techniques significantly reduced pain in the cervical spine and improved Electromyography (EMG) [72]. A considerable improvement in the functional status was observed following the use of a visceral treatment for non-specific low back pain [73,74].

## 6. Visceral Therapy and Physical Activity

Visceral therapy involves the three-dimensional dynamics of the body’s biomechanics: the muscular-skeletal structure, muscular fascial structure, connective tissue and the organs, reflex activity in the central and peripheral nervous system, and also the circulation and draining of fluid systems in the human body [1].

As mentioned above, the objective of visceral therapy for selected dysfunctions is to support movement, articulation and tissue rhythm. Movement is a physiological phenomenon and a fundamental aspect of life. This also applies to micromovements. There is no body activity that is not expressed in the rhythm of pulsations, peristalsis and vibrations. The membranes and the fluids they contain vibrate and transmit mobility to the surrounding structures, which is explained by the tensegration phenomenon [75]. The anatomical structures in the human body (e.g., muscles and internal organs) are not isolated tissue forms. Each of them is surrounded by bands of connective tissue and a system of blood and lymphatic vessels. Treatment of dysfunctions in these structures needs to include the structure of the entire cavity [76]. In the case of dysfunctions in the reproductive system, this involves an assessment of the abdominal and pelvic cavity. For the cavity to be able to maintain its physiological movement, the organs have to move in relation to each other, and also in relation to the surrounding sheaths. There are three pathomechanisms that disturb the sliding motion between organs and the surrounding myofascial structures that can lead to pain and other dysfunctions. These are referred to as pain, changes in the local tissue dynamics, and central sensitization [77].

Dysfunction within an organ irritates C-fiber nerve endings, causing diffuse pain accompanied by increased tension of the skeletal muscles within this organ, which can radiate to areas on the surface of the skin innervated by a particular segment of the spinal cord supplying the organ in question [78]. Pain can occur in any structure connected to the nerve running from that particular spinal segment. This is due to the existence of ganglia transmitting and receiving information to and from the spinal cord through the plexuses [79]. Osteopathic dysfunctions can lead to disproportionate pressure gradients in the pelvic cylinder, which may result in stases, inflammation processes, retentions, visceral disorders and vasomotor restrictions.

Visceral pathologies can manifest themselves in abnormalities related to mobility, motility and the position of organs [80]. Mobility refers to the movement of organs in relation to each other, the diaphragm, the musculoskeletal system, and the cylinder. Organs can cause movement of the intestinal passage, fallopian tubes and ureters, the rhythmic pumping of the heart, as well as lung expansion and deflation. Movement is the determinant of health and life. Motility is the movement of an organ within its area caused by the breathing rhythm, which is why it can change morphologically and take shape in a natural way [76]. It is a sign of an organ’s vitality. The motility of the uterus, ovaries and fallopian tubes shows an upward and dorsal tendency, i.e., upward and backward during the phase of inhalation [81].

The diaphragm, which does not show a propensity for tension, allows the organs to correlate with the movement of inhalation and exhalation, and conditions their physiological sliding motion between each other, the fascias and the cavities [54]. The pelvic urogenital diaphragm should also show similar movement characteristics. If the intra-abdominal pressure is disturbed, the viscera will also yield to compression, and their mobility and motility will be disturbed [82]. According to Andrew Taylor Still, the founder of osteopathy, multiple dysfunctions of the reproductive organs are due to the retention of body fluids. Venous vessels that do not drain arterial blood cause pelvic congestion that results in inflammation, considered by physiotherapists and osteopaths as a functional disorder. For this reason, the objective of treatment is to improve venous drainage within the pelvis [80].

In terms of physiology, the myometrium exhibits contractions that increase during the menstrual cycle and then subsequently decrease. This is a cyclical phenomenon. During the diastole phase, blood rich in oxygen and nutrients enters the tissues, and the relaxation phase takes place. If this process is disturbed, the amount of oxygen decreases and pain occurs. Improper drainage within the small pelvis can lead to extensive tension of the uterus [54].

General procedures for dysfunctions of the reproductive system involve restoration of the postural balance, breathing, pelvic activity, and balancing the pressures between particular diaphragms in the body. It is also essential to pay attention to tension in the muscles within the pelvis and the thoracolumbar fascia, centralization of the hip joint and the area of the sacral bone, and pubic symphysis [53]. An assessment of the spine (particularly at the Th12-L1 and L5-S1 segments) and the sacrococcygeal joint is also important. Restrictions resulting from sympathetic innervation at the thoracolumbar junction of the spine can lead to vasodilation within the small pelvis. The blood pressure decreases, and the supply with oxygen and nutrients deteriorates significantly [54]. We cannot rule out external reasons for dysfunctions of the uterus and the reproductive organs, such as surgical treatments that cause intraperitoneal adhesions within scars, which can also result from the inflammation process [82].

Visceral therapy should begin with a postural analysis, as mobility disorders in the lumbar spine, hip joints and pubic symphysis have a key influence on the dissonance of myofascial tensions. A correct posture is characterized by a symmetry of weight distribution and a vertical line of projection of the center of gravity passing through the acoustic meatus, the acromion, the L3 vertebral body, the greater trochanter and the lateral malleolus. In the anterior projection, the internal organs tend to descend through the inspiratory position of the diaphragm. It is also characterized by extensive tension within the trunk, which results in an inappropriate pressure gradient. The pelvis is tilted forward. The posterior projection features the expiratory position of the diaphragm, a backward tilted pelvis, as well as tension at the level of the sacroiliac joints and the cervicothoracic junction [82].

Visceral therapy helps reduce the experience of pain and simultaneously helps improve women’s quality of life [80,83].

## 7. Conclusions

The use of visceral treatment and physical activity for pain is complementary and also relevant for pregnant women, and may have an effect on reducing oxidative stress in the body and its detrimental effect. Maintaining the mobility and motility of internal organs by means of visceral techniques can positively regulate anatomical relations and physiological processes within the urogenital diaphragm.

The conducted literature review suggests that it is essential to carry out research into the application of visceral therapy in pregnant women.
